# Polymorphisms and gene expression of Notch4 in pulmonary tuberculosis

**DOI:** 10.3389/fimmu.2023.1081483

**Published:** 2023-02-02

**Authors:** Weijun Fang, Hua Liu, Lianhua Qin, Jie Wang, Xiaochen Huang, Sha Pan, Ruijuan Zheng

**Affiliations:** ^1^ School of Public Health, the key Laboratory of Environmental Pollution Monitoring and Disease Control, Ministry of Education, Guizhou Medical University, Guiyang, China; ^2^ Shanghai Key Lab of Tuberculosis, Shanghai Pulmonary Hospital, Tongji University School of Medicine, Shanghai, China

**Keywords:** Mycobacterium tuberculosis, NOTCH4, polymorphism, expression, clinical significance

## Abstract

**Background:**

Tuberculosis (TB) is a serious public health problem to human health, but the pathogenesis of TB remains elusive.

**Methods:**

To identify novel candidate genes associated with TB susceptibility, we performed a population-based case control study to genotype 41SNPs spanning 21 genes in 435 pulmonary TB patients and 375 health donors from China.

**Results:**

We found Notch4 gene rs206018 and rs422951 polymorphisms were associated with susceptibility to pulmonary tuberculosis. The association was validated in another independent cohort including 790 TB patients and 1,190 healthy controls. Moreover, we identified that the rs206018 C allele was associated with higher level of Notch4 in PBMCs from pulmonary TB patients. Furthermore, Notch4 expression increased in TB patients and higher Notch4 expression correlated with the severer pulmonary TB. Finally, we explored the origin and signaling pathways involved in the regulation of Notch4 expression in response to Mycobacterium tuberculosis (Mtb) infection. We determine that Mtb induced Notch4 and its ligand Jagged1expression in macrophages, and Notch4 through TLR2/P38 signaling pathway and Jagged1 through TLR2/ERK signaling pathway.

**Conclusion:**

Our work further strengthens that Notch4 underlay an increased risk of TB in humans and is involved in the occurrence and development of TB, which could serve as a novel target for the host-targeted therapy of TB.

## Introduction

TB, caused by *Mycobacterium tuberculosis*, remains the leading cause of death among infectious diseases throughout the world (1). In 2020, *Mtb* infection was responsible for 9.9 million new cases and 1.3 million deaths (2). Accumulating evidence has demonstrated that host genetic factors contribute to *Mtb* infection outcomes (3–5). However, these factors are extremely intricate and poorly understood.

*Mtb*, as an extremely successful intracellular pathogen, has harbored various host pathways for its survival (6–8). Inhibition of phagosome maturation and autophagy, inhibition of apoptosis and inflammation were the common pathways that were altered by *Mtb*. During *Mtb* infection, effective killing of *Mtb* by the immune cells plays an essential role for the occurrence and development of TB. Dhiraj Kumar and their colleagues performed a genome-wide siRNA screen to find host factors that regulated survival of *Mycobacterium tuberculosis* and identified 275 molecules functionally associated with each other (9). However, the association of these genes with tuberculosis are not fully investigated. The single nucleotide polymorphism (SNP) analysis was a powerful tool for identifying susceptibility genes associated with diseases and candidate gene approach was a method that was important to detect genetic susceptibility to diseases. To identify novel genes associated with TB susceptibility, we selected 21 genes as candidate genes for TB from 275 screening genes to perform SNP analysis. The 21 candidate genes are involved in the several pathways including inflammatory response through extracellular pathway (IFNB1, CXCL5, CCL11, CXCL14, LTA4H) and intracellular/cellular pathway (NLRC4, NLRP1, IRAK2, IRF-4, LCN2, CARD9, ARRB2, NOTCH4), apoptosis (FASLG, PDCD1LG2, TRAF6, NOD1, TNFSF15, RIPK2), and Th2 response (IL-4, GATA3).

Tag-SNP is the representative SNP in a region of the genome with high linkage that represents a group of SNPs in a haplotype block. Tag SNP is often performed for candidate gene association studies using HapMap and gene resequencing data (10). Using tag SNP approach, we can analyze the association between genetic polymorphism and phenotypes without genotyping every SNP in a chromosomal region, reducing the detection time and cost. The previous researches have shown that tag SNP was a power tool for genetic study.

In this study, we selected 41 tag SNPs spanning these 21 candidate genes to investigate whether the SNPs in these genes were associated with susceptibility to TB. We performed a population-based case-control screening and identified Notch4 as a susceptible gene for TB. Furthermore, Notch4 expression was significantly elevated in TB patients, and higher Notch4 expression was associated with severer TB.

## Materials and methods

### Clinical samples

Two case-control cohorts were used in this study to investigate the association between Notch4 polymorphisms and susceptibility to TB. All subjects were genetically unrelated members of the Chinese Han population. All protocols were approved by the local ethics committee of Shanghai Pulmonary Hospital and the signed informed consent was obtained from all subjects (permit number: K19-054Y).

The clinical presentation and radiological signs (such as X-ray or computed tomography (CT) scan) were the diagnostic criteria for TB, and confirmed by sputum culture positivity. All TB patients was negative of anti-human immunodeficiency virus (HIV) antibody. Average age was 48 years and 71% of patients were male. The physical examination donors, with no history of previous TB or anti-mycobacterial treatments and no evidence of TB-related infiltration in chest X-rays, were collected as control group. Average age was 45 years and 68% of controls were male. The controls in our study were recruited from a pool of individuals who participated in the health examination program. These controls were living mainly in the same geographical area as the cases. The samples of screening stage included 435 TB patients and 375 healthy controls, which have been used in our previous study (11). The samples of replication stage included 790 TB patients and 1190 health controls, which have been used in our previous TB genome-wide association studies (GWAS) (5). We performed a *post-hoc* power analysis by G.power 3.1 software for genetic analysis using our current sample sizes. when the α was 0.05, effect size was 0.2, sample size group1was 375, sample size group2 was 435, the power was 0.8.

For validation of association between genetic variation and Notch4 mRNA expression, 40 PTB patients and 54 healthy controls were recruited from the Nantong Sixth people’s hospital. For nucleic acid extraction, two tubes of whole blood samples were collected from each donor, and one sample was for DNA extraction and another sample was for isolation of peripheral blood mononuclear cells (PBMC) for RNA extraction.

For detection of Notch4 mRNA expression, the whole blood samples from 84 TB patients and 53 healthy controls were recruited. For analysis of association between Notch4 expression and clinical indices, the clinical data from 84 TB patients mentioned above were collected, including status of patients with initial treatment or retreatment, the presence of mycobacteria in the sputum smear, the presence of cavity in the lung and ESR. The samples information of demographic and characteristics were listed in **Supplementary Table 1**. For evaluation the notch4 protein expression, lung tissue from 5 TB patients with pulmonary lobectomy were investigated.

### Genomic DNA extraction

The 2 ml ethylenediaminetetraacetic acid (EDTA)-treated blood were collected and 200ul of blood sample were used to extract genomic DNA by QIAamp DNA Blood Mini Kit (Qiagen GmbH, Germany) according to manufacturer’s instructions.

### SNP selection

We selected 41 tag SNPs for these 21 candidate genes. Haploview V4.2 was employed to run the tag SNPs. For each gene, tag SNPs covered 2000 base pairs (bp) upstream of the annotated transcription start site and downstream at the end of the last exon were selected. In addition, a threshold of r^2^, HardyeWeinberg *p* value and minor allele frequency was greater than or equal to 0.8,0.01 and 0.1 respectively.

### Genotyping and quality control

The 41 tag SNPs genotyping work was done by the Shanghai BioWing Applied Biotechnology Company (http://www.biowing.com.cn/) using ligase detection reactions (LDR) as described previously (12). In brief, multiplex PCR was used to amplify DNA sequences of each subject. After the completion of amplification, the ligation reaction for each subject was carried out. The LDR were performed and LDR results were analyzed with Genemapper software. The data of rs422951 and rs206018 in replication stage were obtained from our previous TB GWAS and genotyping using Affymetrix Axiom CHB arrays. An Affymetrix Axiom^®^ Genome-Wide CHB1& CHB2 Array Plate Set (two chips/set) was used for genotyping (5). SNPs deviated significantly from Hardy-Weinberg equilibrium (*p*≤ 0.001) in controls were excluded.

### Mice

*Tlr2^−/−^*, *Tlr3^−/−^* and *Tlr4^−/−^* mice were originally from Professor S. Akira (Osaka University, Osaka, Japan). C57BL/6 mice were purchased from Shanghai Laboratory Animal Center (Shanghai, China). All mice were bred in specific pathogen-free (SPF) conditions at the Laboratory Animal Center of Tongji University. 6~8 weeks old female mice were used. Animal procedures were approved by the Animal Experiment Administration Committee of Shanghai Pulmonary Hospital (K19-054Y).

### Reagents and antibodies

SB203580, SP600125, PD98059 and PDTC were purchased from Calbiochem and used at concentration of 10 μM. Rabbit anti-Notch4 (sc-5594, 1:200 dilution) were purchased from Santa Cruz Biotechnology (Santa Cruz, CA, USA); Rabbit anti-Jagged1 (2620, 1:000 dilution) from Cell Signaling Technology; Rabbit anti-GAPDH (SAB2701826) from Sigma. PGN were purchased from Sigma.

### Peripheral blood mononuclear cells isolation

The density gradient centrifugation method was used for isolation of PBMCs from EDTA treated whole blood by Ficoll separation (Ficoll-Paque plus; Amersham Biosciences). After twice washes in PBS, PBMCs were subjected to further analyses.

### Mouse peritoneal macrophage isolation and infection

Peritoneal macrophages were isolated as previously described (11). Briefly, mice were injected intraperitoneally (IP) with 2.0 ml of 4% Brewer’s thioglycollate medium (Sigma-Aldrich). The primary macrophages were collected after 3 days and were plated in 12-well plates at 10^6^ cells/well in culture media of RPMI 1640 (GIBCO) supplemented with 10% heat-inactivated fetal bovine serum (Sigma-395 Aldrich, F0804), penicillin/streptomycin, and incubated at 37°C at 5% CO_2_.

*Mycobacterium bovis* BCG and *Mtb* H37Rv were grown to mid-log phase in Middlebrook 7H9 medium (Becton Dickinson, Cockeysville, MD) with 0.05% Tween-80 and 10% oleic acid-albumin-dextrose-catalase (OADC) enrichment (Becton Dickinson, Sparks, MD). Before infection, *Mycobacteria* were suspended in complete medium without antibiotics. The macrophages were infected with H37Rv or BCG at MOI of 5.

### Aerosol infection of mice

All animal study protocols were reviewed and approved by Tongji University School of Medicine review boards for animal studies. Infection studies were carried out as previously described (13). Briefly, mice were infected with *Mtb* H37Rv with ~200 CFU using a Glas-Col inhalation exposure system (Glas-col Inc., Terre Haute, Ind) for four weeks.

### Immunoblot analysis

Cells were lysed with RIPA lysis buffer, followed by centrifugation at 12,000 g at 4°C for 10 min, and the supernatants were collected and then boiled in SDS loading buffer. The proteins were resolved by PAGE and then transferred to a PVDF membrane. ECL reagent (Thermo Scientific) was applied for immunoblot analysis.

### Immunohistochemistry

Segments of lung tissue, from 5 TB patients with pulmonary lobectomy and 6 *Mtb*-infected mice, were fixed in 10% buffered formalin and embedded in paraffin. The immunohistochemistry staining with Notch4 antibody was at a 1: 250 dilution. After counterstaining in hematoxylin, sections were mounted and examined. Studies on clinical samples were conducted in accordance with ethical guidelines of the institutional review board of Tongji University.

### Quantitative real-time PCR

The PBMCs of clinical samples, macrophages and lungs of mice were extracted total RNA with TRIzol reagent according to the manufacturer’s instructions (Invitrogen). Next, 1 μg of total RNA was reverse transcribed using the ReverTra Ace^®^ qPCR RT Kit (Toyobo, FSQ-101). A SYBR RT-PCR kit (Toyobo, QPK-212) were used for quantitative real-time PCR analysis. The relative mRNA expression of different genes was calculated by comparison with the control gene Gapdh (encoding GAPDH) using the 2^-△△Ct^ method. The sequences of primers for qPCR were shown in **Supplementary Table 2**.

### Statistical analysis

Hardy-Weinberg equilibrium (HWE) of each polymorphism was determined by the program HWE. The significant differences of allele and genotype frequencies were calculated using Pearson’s χ2 test by SPSS soft (SPSS Inc, Chicago, IL). For the results of gene expression, the statistical significance between two groups were determined by Student *t*-test or Mann-Whitney *U* test using Graph-Pad Prism software (version 5.0). If the variance is homogeneous, Student t-test is used, and if the variance is not homogeneous, Mann-Whitney U test is used. The correlation between the Notch4 expression and ESR was determined by Spearman. *P<0.05 was considered statistically significant.

## Results

### Notch4 gene polymorphism is associated with TB genetic susceptibility

To identify new TB susceptible genes, we performed a genetic screening of 21 candidate genes in 435 pulmonary TB patients and 375 healthy controls, the allelic frequencies of 41tag SNPs among these genes were analyzed. The results showed that the allele frequencies of Notch4 SNP rs422951 and rs206018 were statistically difference between the TB and control subjects. While p-values became no-significant after adjusting, suggesting they are suggestive association with TB susceptibility (**Supplementary Table 3**). Further analysis was showed that the genotype frequency of rs422951 and rs206018 was significantly different between TB patients and healthy controls (rs422951, P=0.035, and rs206018, P=0.026, **Table 1**) and rs422951G and rs206018G allele were associated with protection against TB due to odds ratio was less than 1 (rs422951, OR=0.725, *P*=0.012, and rs206018, OR=0.777, *P*=0.047, **Table 1**). The analysis of genetic models found the strongest association for rs422951 was a dominant mode (odds ratio [OR] =0.679; P=0.01, **Table 1**). In contrast, SNP rs206018 was a recessive model (OR=0.452; P=0.007, **Table 2**).

**Table 1:** Genotype and Allele distribution and frequencies of Notch4 gene rs422951and rs206018 in pulmonary tuberculosis patients and controls.

dbSNP	Genotype/Alleles	Genotype frequency		*P* value	OR(95% CI)
		TB patients, N (%)	Controls, N (%)		
Discovery					
rs422951	A/A	303(69.98)	223(61.26)	0.035	
	A/G	117(27.02)	126(34.62)		
	G/G	13 (3.00)	15 (4.12)		
	A	723(83.49)	572(78.58)		
	G	143(16.51)	156(21.42)	0.012	0.725(0.564~0.933)
rs206018	C/C	257(59.63)	209(55.73)	0.026	
	C/G	156(36.19)	133(35.47)		
	G/G	18 (4.18)	33 (8.80)		
	C	670(77.72)	551(73.47)		
	G	192(22.28)	199(26.53)	0.047	0.777(0.619~0.976)
Replication					
rs422951	A/A	541(69.28)	743(67.73)	0.013	
	A/G	220(28.17)	350(28.93)		
	G/G	20(2.55)	56(3.34)		
	A	1302(83.35)	1836(79.90)		
	G	260(16.65)	462(20.10)	0.007	0.794(0.671~0.939)
rs206018	C/C	358(46.98)	544(44.02)	0.000064	
	C/G	371 (48.68)	524 (44.33)		
	G/G	33(4.34)	114(9.65)		
	C	1087(71.33)	1612(68.19)		
	G	437(28.67)	752(31.81)	0.038	0.862(0.749~0.992)

CI, confidence interval; OR, odds ratio.

Hardy Weinberg (H-W) equilibrium was calculated from controls only. P value of rs422951 and rs206018 was 0.594 and 0.08 for discovery stage, and 0.079 and 0.452 for replication stage in controls.

P-values of genotype calculated by 2x3 contingency Table; P-values of allele calculated by 2x2 contingency Table.

**Table 2:** Distribution and frequencies of Notch4 genotype with different genetic models in tuberculosis patients and controls.

	Inherited model	Genotype	TB	controls	OR	95% CI	*P* Value
**Discovery**	rs422951						
	Dominant	A/G and G/G	130	141	0.679	0.505~0.911	0.01
		A/A	303	223			
**Replication**	Dominant	A/G and G/G	240	406	0.812	0.669~0.986	0.035
		A/A	541	743			
**Discovery**	rs206018						
	Recessive	G/G	18	33	0.452	0.250~0.816	0.007
		C/G and C/C	413	342			
**Replication**	Recessive	G/G	33	114	0.424	0.285~0.632	0.000015
		C/G and C/C	729	1068			

P-values of allele calculated by 2x2 contingency Table.

The association of rs422951 and rs206018 with TB was replicated in an independent cohort including 790 TB patients and 1,190 healthy controls. The results were showed the polymorphism of rs206018 and rs422951were associated with genetic susceptibility to TB (**Table 1**). The allelic frequencies for rs422951G and rs206018G were lower in TB patients compared with controls, indicating that G allele of rs422951 and rs206018 reduced the risk of developing TB (**Table 2**). Thus, the Notch4 SNP rs422951 and rs206018 was associated with genetic susceptibility to TB and the two SNPs played a resistant role.

### Genetic variation of Notch4 is associated with its expression

**Figure 1:**
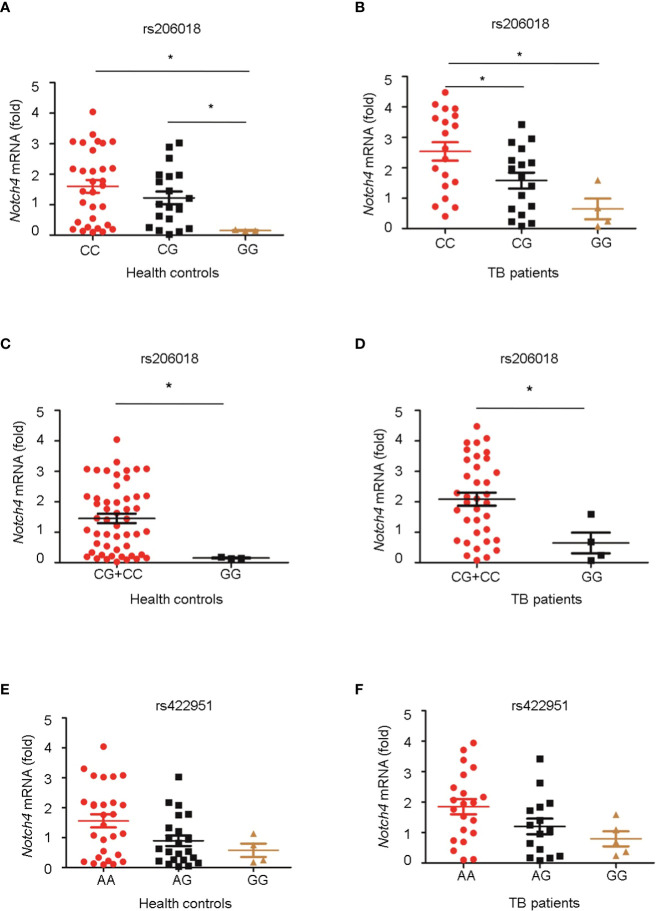
Association of Notch4 polymorphisms with its mRNA expression. Genomic DNA and mRNA were isolated from PBMC of healthy individuals and TB patients. *Notch4* mRNA level was measured and normalized to *gapdh* gene. The genotypes of two *Notch4* polymorphisms were examined for association with *Notch4* mRNA expression. **(A, B)** Association between *Notch4* SNP rs206018 and *Notch4* mRNA level in healthy controls **(A)** and in TB patients **(B)**. **(C, D)** Notch4 SNP rs206018 affects Notch4 mRNA expression in a recessive model in health controls **(C)** and in TB patients **(D)**. **(E, F)** Association between Notch4 SNP rs422957 and Notch4 mRNA level in healthy controls **(E)** and in TB patients **(F)**. Difference between two groups were analyzed by Mann-Whitney *U* test due to the variance was not homogeneous, *p < 0.05.

Next, we genotyped the rs422951 or rs206018 polymorphisms in 54 healthy controls and 40 TB patients and examined the association between their genetic variations and *Notch4* mRNA expression in PBMC. The minor homozygote of SNP rs206018 was significantly associated with decreased *Notch4* mRNA expression compared to major homozygote in both healthy controls (**Figure 1A**) and TB patients (**Figure 1B**). This association was also significant in a recessive model; the GG genotype was associated with lower levels of *Notch4* mRNA than the GC+CC genotype in healthy controls (**Figure 1C**) and in TB patients (**Figure 1D**). However, no significant association of SNP rs422951 with *Notch4* mRNA expression was observed (**Figures 1E, F**). These data suggested that the rs206018C allele might up-regulate Notch4 mRNA expression.

### 4 Expression Levels of Notch4 Increase in the TB Patients

**Figure 2:**
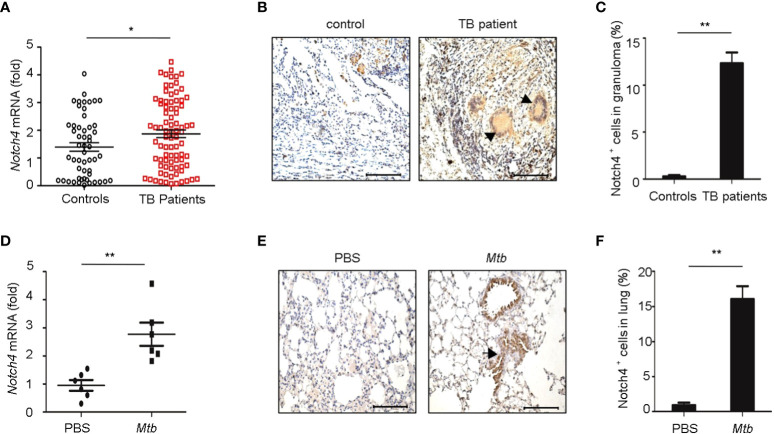
Elevated Notch4 expression in tuberculosis patients. **(A)** qRT-PCR detection of Notch4 expression in PBMCs from 53 healthy individuals and 84 TB patients. **(B, C)** Representative immunostaining of Notch4 in granulomas from 5 TB patients **(B)** and quantification of Notch4 positive cells **(C)**. Bar, 100 µm. Magnification: ×200. **(D)** qRT-PCR of *Notch4* mRNA in lungs of mice aerosol-infected with ~200 CFU *Mtb* H37Rv for 4 weeks (6 mice/group). **(E, F)** Representative immunostaining of Notch4 in lungs of mice infected as in E **(E)** and quantification of Notch4 positive cells **(F)**. Bar, 100 µm. Magnification: ×200. The representative images were shown **(B, E)**. Results shown in **(A, C, D, F)** were expressed as mean ± SEM by the Student *t* test, *P < 0.05 and **P <0.01.

To investigate the expression levels of Notch4 in pulmonary tuberculosis, we first explored Notch4 expression in PBMCs from TB patients. Compared with healthy controls, the Notch4 mRNA expression was significantly upregulated in TB patients (**Figure 2A**). We further detected Notch4 expression in granulomas of tuberculosis. The lung tissue sections surgically removed from TB patients were analyzed by immunohistochemistry with anti-Notch4 antibodies, and normal tissue next to the granulomas of TB patients as a control. The results showed that Notch4 was expressed in the granuloma of TB patients and was mainly localized in inflammatory infiltrates (**Figures 2B, C**). Similarly, the expression level of Notch4 was also significantly increased in the lungs of mice infected with *Mtb* H37Rv (**Figures 2D- F**). Thus, these results demonstrated that Notch4 might be involved in TB pathogenesis.

### 4 Higher Notch4 Expression is Associated With Severer TB

**Figure 3:**
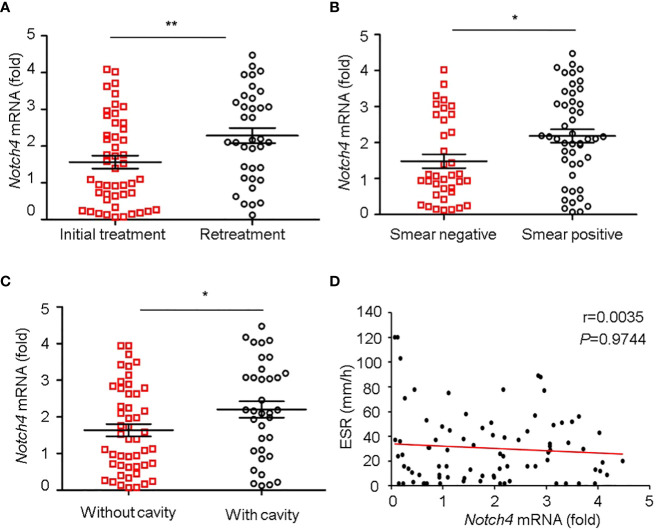
Correlation between Notch4 expression and clinical characteristics. **(A-D)** qRT-PCR detection of Notch4 expression in PBMCs from 84 TB patients and analysis the correlation between Notch4 expression and the status of patients with initial treatment or retreatment **(A)**, bacillary load in sputum **(B)**, lung field with cavity or without cavity **(C)**, and erythrocyte sedimentation rate **(D)**. The data were expressed as mean ± SEM by the Student *t* test, *P <0.05 and **P <0.01.

We further investigated the relationship between Notch4 expression and clinical characteristics of TB patients. We observed that the level of Notch4 was significantly higher in patients with retreatment than in patients with initial treatment (**Figure 3A**), suggesting that upregulation of Notch4 might contribute to decrease immune function against *Mtb* infection. We also found that patients with sputum positive or patients with lung cavity had higher Notch4 expression than those of patients with sputum negative (**Figure 3B**) or without lung cavity (**Figure 3C**), suggesting that notch4 might be involved in the progression of TB. No correlation of Notch4 expression with ESR was observed in TB patients (**Figure 3D**). Taken together, these results indicated that higher Notch4 expression is associated with severer pulmonary TB.

### *Mtb* induced Notch4 expression in macrophages

**Figure 4:**
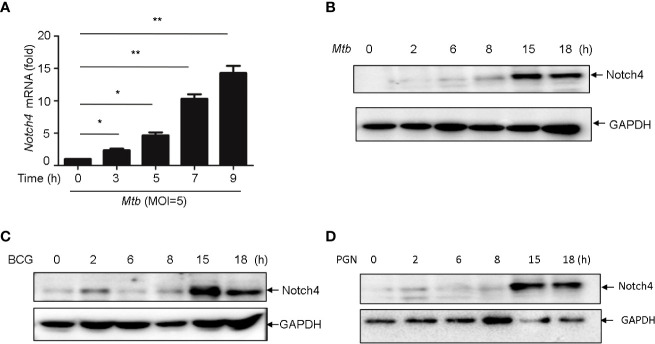
*Mycobacterium* induced Notch4 expression in Macrophages. **(A)** qRT-PCR detection of Notch4 expression in peritoneal macrophages of mice infected with *Mtb* for indicated times. **(B-D)** IB detection of Notch4 expression in peritoneal macrophages of mice infected with *Mtb* **(B)**, BCG **(C)** and PGN (25 μg/ml) **(D)** for the indicated times. MOI=5. Results shown in **(A)** was expressed as mean ± SEM and the representative of three independent experiments. *P < 0.05 and **P < 0.01 by the Student *t* test. Western blots were representative of three independent experiments.

In TB, macrophages play an important role in host defense against *Mtb.* To determine whether macrophage is one of the origin of Notch4 in response to mycobacterial infection, we detected Notch4 expression in murine primary peritoneal macrophages (PM) infection with H37Rv. The results showed that Notch4 mRNA level was significantly enhanced when these cells were infected with H37Rv (**Figure 4A**). Moreover, NOTCH4 protein level was also induced by the infection with either H37Rv (**Figure 4B**) or BCG (**Figure 4C**) in PM. To test whether *Mtb*-induced Notch4 expression in macrophages depends on peptidoglycan (PGN), a core of *mycobacterium* cell wall, we detected Notch4 expression in macrophages treated with PGN. The results showed that PGN markedly increased NOTCH4 protein production (**Figure 4D**). Taken together, sensing of mycobacteria involving PGN by macrophage led to the induction of Notch4, which at least partially contributed to the enrichment of Notch4 in the process of *Mtb* infection.

### Induction of Notch4 by *Mtb via* TLR2/p38 pathway in macrophages

**Figure 5:**
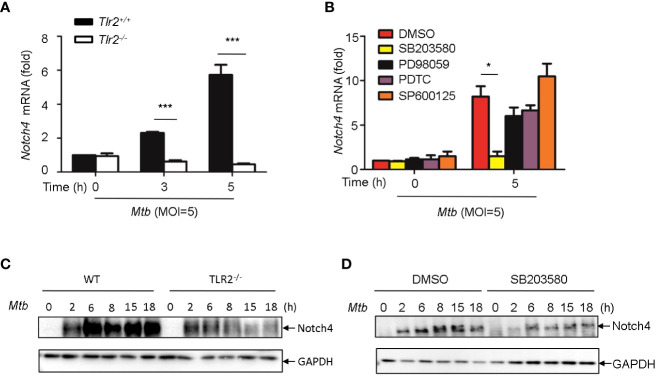
Induction of Notch4 by *Mtb via* TLR2-p38 signaling pathway. **(A)** qRT-PCR detection of Notch4 expression in *Mtb*-infected peritoneal macrophages isolated from WT or *Tlr2* ^-/-^ mice for indicated times. **(B)** qRT-PCR detection of Notch4 mRNA in mice peritoneal macrophages pretreated with ERK inhibitor PD98059 (10 uM), NF-κb inhibitor PDTC (10uM), JNK inhibitor SP600125 (10uM) and p38 inhibitor SB203580(10uM) for 1 h before *Mtb* infection. **(C)** IB detection of Notch4 expression in peritoneal macrophages from WT or *Tlr2* ^-/-^mice infected with *Mtb*. **(D)** IB of Notch4 in *Mtb*-infected mice peritoneal macrophages pretreated with SB203580 for 1 (h) MOI=5. Data shown in **(A, B)** are Mean ± SEM and the representative of three independent experiments. *P < 0.05 and *** *P* < 0.001 by the Student’s *t* test. Western blots were representative of three independent experiments.

It has been reported that PGN activates cells through the Toll-like receptor 2 (TLR2) (14, 15), we therefore investigated whether *Mtb*-induced Notch4 expression in macrophages by engaging TLR2 receptor. We detected Notch4 expression in macrophages isolated from *Tlr2* deficient mice infected with H37Rv. Compared with macrophages from wild type mice, *Mtb*-induced Notch4 mRNA expression was markedly reduced in *Tlr2* ^-/-^ macrophages (**Figure 5A**). To further investigate the signaling pathways involved in the induction of Notch4 by *Mtb*, macrophages were infected with *Mtb* in the presence of different inhibitors targeting ERK (PD98059), JNK (SP600125), p38 (SB203580) or NF-κB (PDTC). Specific inhibition of the p38 pathway by SB203580 markedly reduced *Mtb*-induced *Notch4* mRNA expression in macrophages (**Figure 5B**). *Tlr2* deficiency or treatment of SB203580 also markedly reduced *Mtb* induced NOTCH4 protein production (**Figure 5C, D**). These data indicated that TLR2/p38 signaling pathway involved in the *Mtb* induced Notch4 expression.

### Induction of jagged1 by *Mtb* in macrophage *via* TLR2/ERK signaling

**Figure 6:**
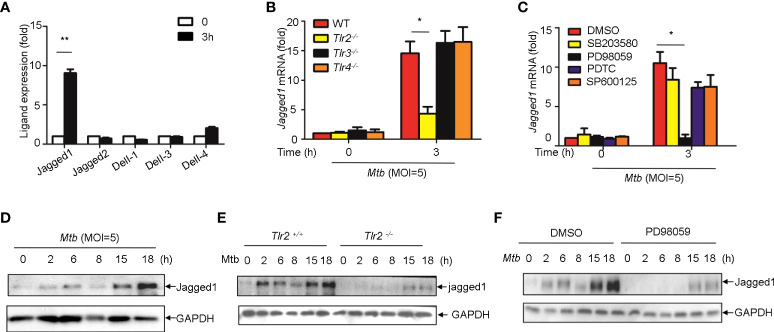
*Mtb* up-regulate Jagged1 expression in macrophage *via* TLR2-triggered ERK signaling. **(A)** qRT-PCR detection of different Notch ligands expression in *Mtb*-infected peritoneal macrophages. **(B)** qRT-PCR detection of *Jagged1* mRNA in WT, *Tlr2^-/-^, Tlr3^-/-^*, *Tlr4^-/-^* macrophages infected with *Mtb* for 3h. **(C)** qRT-PCR detection of Jagged1 mRNA in *Mtb*-infected mice peritoneal macrophages pretreated with ERK inhibitor PD98059 (10 uM), NF-κb inhibitor PDTC (10uM), JNK inhibitor SP600125 (10uM) and p38 inhibitor SB203580(10uM) for 1 (h) **(D)** IB detection of Jagged1 in *Mtb*-infected macrophages. **(E)** IB detection of Jagged1 expression in peritoneal macrophages of WT or *Tlr2* ^-/-^mice infected with *Mtb* for indicated times. **(F)** IB detection of Jagged1 in *Mtb*-infected macrophages treated as in **(C, G)** Diagram of Notch4 and jagged1 expression. MOI=5. Data shown in **(A-C)** are Mean ± SEM and the representative of three independent experiments. *P< 0.05, **P< 0.01 by the Student’s *t* test. Data in **(D-F)** were representative of three independent experiments.

We next sought to examine whether Notch ligands were also induced by *Mtb*. Of the five mammalian Notch ligands, the mRNA level of *Jagged1* was significantly increased in macrophages infected with *Mtb* (**Figure 6A**). *Tlr2* but not *Tlr3* or *Tlr4* deficiency markedly attenuated the induction of *Jagged1* expression by *Mtb* (**Figure 6B**). Inhibition of ERK by selective inhibitor PD98059 suppressed *Mtb*-induced *Jagged1* mRNA expression (**Figure 6C**). Consistently, *Mtb*-induced expression of *Jagged1* was further validated at the protein level using an immunoblot assay (**Figure 6D**). Deletion of *Tlr2* or specific inhibition of ERK by PD98059 markedly reduced *Mtb*-induced production of Jagged1 (**Figures 6E, F**), suggesting an important role for the TLR2-ERK pathway in Jagged1 induction.

## Discussion

**Figure 7:**
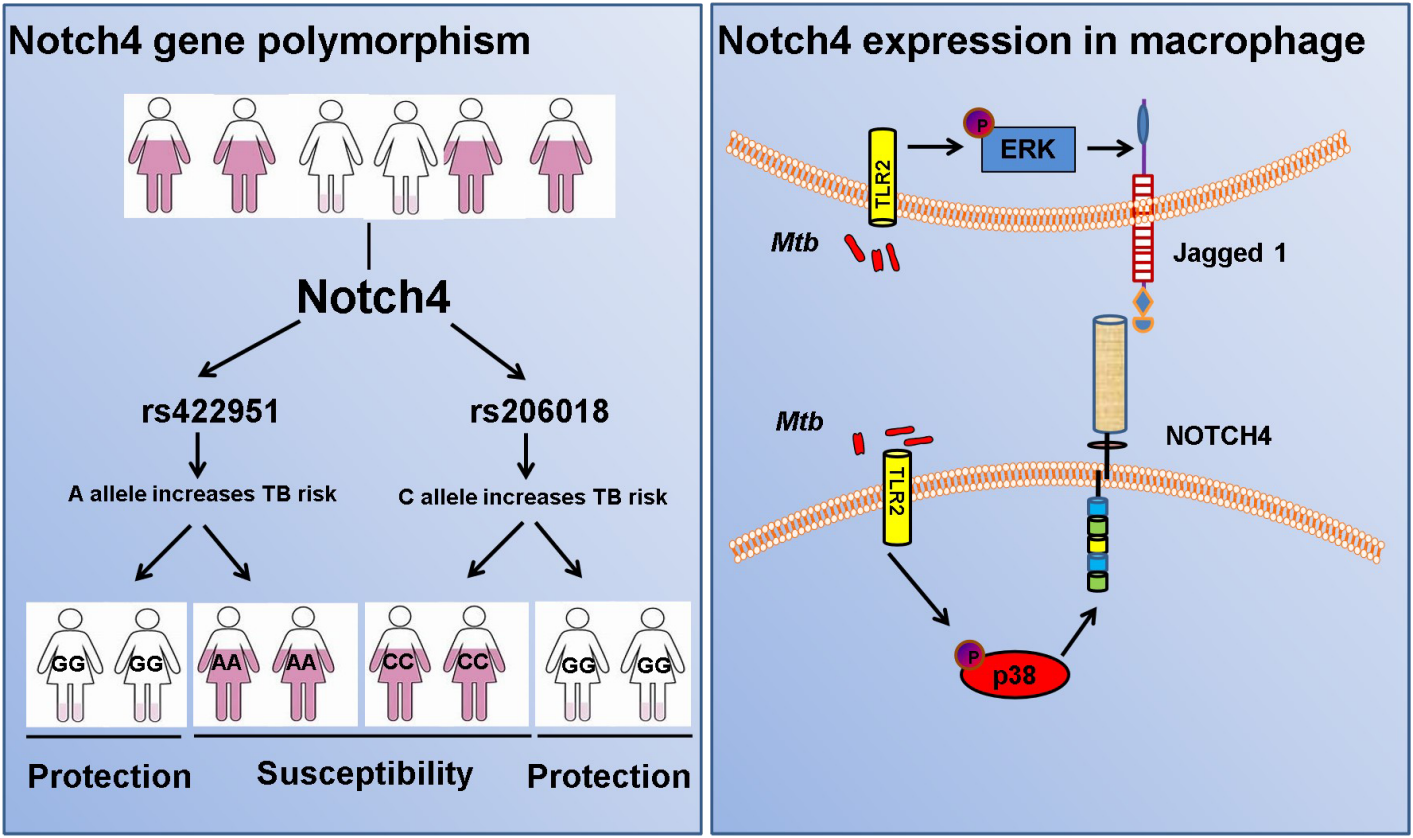
Diagram of Notch4 polymorphisms and gene expression.

Here, we performed a two-stage case-control study in 2790 individuals from China to identify novel candidate genes for susceptibility to tuberculosis. Our findings demonstrated Notch4 was a susceptibility gene for TB and genetic variation of Notch4 is associated with its expression. Notch4 expression was significantly increased in TB patients and associated with severer pulmonary TB. In addition, mycobacteria triggered the expression of Notch4 and its corresponding ligand jagged1 in macrophages through TLR2 signaling pathway (**Figure 7**). Our findings here demonstrate that naturally occurred polymorphism and reduced expression in Notch4 decrease the risk of developing active TB in humans. Our previous study reported that Notch4 negatively regulates the inflammatory response and Notch4-deficient mice are more resistant to Mtb infection (13). Together, these experimental and clinical studies highlight a pivotal role for Notch4 signaling in determining the outcome of mycobacterial infection.

Notch4, a member of the Notch receptor family in mammals, was originally identified as a viral oncogene Int3 in mice, and is involved in the initiation of mammary tumors (16, 17). Several other studies reported that Notch4 gene polymorphisms were associated with susceptibility to a number of diseases (18–22), suggesting that Notch4 was an important genetic factor. Here, we found G allele of rs422951 had a lower frequency in TB patient and was related with resistance to TB. Variant rs422951 is a nonsynonymous SNP and A allele replaced by G allele leads to substitution of Threonine with Alanine at the sixth exon at codon 320 of Notch4 gene (ACC to GCC, T320A). Interestingly, Jingwei Zhang and his colleagues also found Notch4 missense mutation rs422951 was associated with susceptibility to tuberculosis in Chinese population and the G variants of rs422951 conferred protective factors in TB susceptibility (23). Other studies have identified this variant involved in autoimmune and infectious diseases. In the Japanese population, rs422951 was conferred resistance to multiple sclerosis (21, 24). A genome-wide association study (GWAS) in Chinese population indicated rs422951 was a chronic hepatitis B susceptibility locus (25). This locus has also been confirmed to be related to the susceptibility of occasional inclusion body myositis (26) and hepatitis B virus-related hepatocellular carcinoma (27), suggesting that rs422951 was an important genetic factor.

Our study also indicated that the Notch4 SNP rs206018 was genetically associated with susceptibility to TB and rs206018 C allele was associated with higher level of Notch4. Bioinformatics analysis by HaploReg soft showed rs206018 was located in the intron region of Notch4 gene and was also an expression Quantitative Trait Locus (eQTL) SNP for Notch4 gene (data not show), suggesting this variant exerted allele-specific effects on Notch4 expression. Therefore, we are tempting to speculate that the mutation of rs206018G is likely to regulate Notch4 gene expression, which might potentially make the individuals with GG genotype more resistant to *Mtb* infection. In addition, Since the genotyping data of replication were retrieved from our previous TB GWAS, methodological problems might have some influence on the results of the rs206018 genotype and allele distribution and it is better to verify the result in larger samples or other populations in further study.

There is accumulating evidence that aberrant Notch4 expression has a critical role in tumorigenesis (28, 29). However, Notch4 expression level and clinical significance in tuberculosis remains uncertain. In present study, we observed an elevation and enrichment of Notch4 in the lung of both TB patients and *Mtb*-infected mice. We also found the patients with a higher abundance of Notch4 in PBMCs had a higher amount of *Mtb* in sputum. The correlation of Notch4 expression levels and amount of sputum bacteria in TB patients suggested that differences in Notch4 expression might lead to divergent ability of elimination *Mtb* and different disease outcomes. It has been reported the increase in Notch1 expression in T cells and DLL4 expression in intermediate monocytes were related to severe forms of TB (30). Our previous studies have been shown that the expression of Notch4 was reversely correlated with production of IL-6 in PBMCs of TB patients (13). Taking together, enrichment of notch4 might exacerbate mycobacterial infection and Notch4 expression may be considered as a potential clinical indicator.

We further explored the source of Notch4 during *Mtb* infection. We observed an elevation and enrichment of Notch4 might partially arise from mycobacteria-induced Notch4 expression in macrophages. Our results were shown PGN, a pathogen associated molecular patterns (PAMPs) on the cell wall of *Mtb*, was recognized by TLR2 on macrophages, and activated the p38 signaling pathway to induce notch4 production. Main knowledge about the role of TLR signaling in Notch expression is derived from studies of Notch1 and Notch2, the most conserved paralogs. Several studies have shown that the stimulation of TLR ligands enhances the expression of Notch1/2, Notch ligands Jagged1 and its target genes (31–33). In this study, we demonstrated that *Mtb* infection induced the expression of Notch4 and Jagged1 *via* TLR signaling pathway. In our previous study, we found Notch4 inhibited *Mtb*-triggered production of proinflammatory cytokines *via* TLR signaling pathway (13). Taking together, regulation of cross-talk between Notch and TLR signaling pathways appears to be a novel mechanism that could be used by *Mtb* as a strategy to evade the host immune responses against them.

In conclusion, Notch4 is involved in the occurrence and development of tuberculosis. In the case of tuberculosis infection, people carrying the C allele in rs206018 can produce more Notch4 through immune cells dominated by macrophages, and Notch4 can weaken the ability of the host to resist Mtb infection to lead to greater susceptibility to TB and more severe clinical manifestations than individuals with the G allele. Further studies are warranted to investigate the functional characteristics of these genetic variants of Notch4 and their effect on *Mtb* infection.

## Data availability statement

The datasets presented in this article are not readily available because of legal restrictions. Requests to access the datasets should be directed to the corresponding author.

## Ethics statement

The studies involving human participants were reviewed and approved by ethics committee of Shanghai Pulmonary Hospital. The patients/participants provided their written informed consent to participate in this study. The animal study was reviewed and approved by Animal Experiment Administration Committee of Shanghai Pulmonary Hospital. Written informed consent was obtained from the owners for the participation of their animals in this study.

## Author contributions

RZ, designed experiments. WF, HL, JW, LQ, SP, and RZ performed experiments and analyzed data. JW, XH facilitated sample collection and transport. WF, HL, and XH contributed to mouse experiments. WF, and RZ, prepared the figures and wrote the manuscript. All authors contributed to the article and approved the submitted version.
