# Supplementary material for: Polymorphisms and gene expression of Notch4 in pulmonary tuberculosis
Source: Front Immunol. 2023 Feb 2;14:1081483. doi: 10.3389/fimmu.2023.1081483 (PMC9933242; doi:10.3389/fimmu.2023.1081483)
Supplement: Supplementary file 1 [file Table_1.docx]

**Polymorphisms and gene expression of Notch4 in pulmonary tuberculosis**

Weijun Fang^1,2^^†^, Hua Liu^1,2†^, Lianhua Qin^2^, Jie Wang^2^, Xiaochen Huang^2^, Sha Pan^1^, Ruijuan Zheng ^1,2*^

^1^ School of Public Health, the key Laboratory of Environmental Pollution Monitoring and Disease Control, Ministry of Education, Guizhou Medical University, Guiyang 550025, China

^2^ Shanghai Key Lab of Tuberculosis, Shanghai Pulmonary Hospital, Tongji University School of Medicine, Shanghai 200043, China

^†^Contributed equally to this work

*Correspondence should be addressed to R.J. Z. ([zhruijuan923@163.com](mailto:zhruijuan923@163.com))

Supplementary Table 1-3

**Supplementary Table 1. The information of clinical samples**

|  | | **Characteristic** | **TB, n (%)** | **Control, n (%)** | ***P* value** |
| --- | --- | --- | --- | --- | --- |
| Genotyping assay | **Sex** | |  |  |  |
|  | Male | | 870(71.02) | 1064(67.99) | 0.085 |
|  | Female | | 355(28.98) | 501(32.01) |  |
|  | **Age (years)** | |  |  |  |
|  | Average | | 48.33±11.11 | 45.21±9.84 |  |
|  |  | |  |  |  |
| Genetic variation and expression experiment | **Sex** | |  |  |  |
|  | Male | | 27(67.5) | 33(61.11) | 0.524 |
|  | Female | | 13(32.5) | 21(38.89) |  |
|  | **Age (years)** | |  |  |  |
|  | Average | | 51.03±12.08 | 49.15±14.16 |  |
|  |  | |  |  |  |
| mRNA experiment | **Sex** | |  |  |  |
|  | Male | | 46(54.76) | 31(58.49) | 0.668 |
|  | Female | | 38(45.24) | 22(41.51) |  |
|  | **Age (years)** | |  |  |  |
|  | Average | | 49.133±12.11 | 46.56±8.28 |  |

**Supplementary Table 2. Primer sequences**

| qPCR primers |  |
| --- | --- |
| mGAPDH F | 5'CCCACTAACATCAAATGGGG3' |
| mGAPDH R | 5'CCTTCCACAATGCCAAAGTT3' |
| mNotch4 F | 5'CTCTTGCCACTCAATTTCCCT3' |
| mNotch4 R | 5'TTGCAGAGTTGGGTATCCCTG3' |
| mJagged1 F | 5'GAAGTCAGAGTTCAGAGGCGTCC3' |
| mJagged1 R | 5'AGTAGAAGGCTGTCACCAAGCAAC3' |
| mJagged2 F | 5'TGCTGTGGAGGTGGCTATGTCT3' |
| mJagged2 R | 5'TGTTTCCACCTTGACCTCGGT3' |
| mDell-1 F | 5'GGACCTCATGAGGCATATGG3' |
| mDell-1 R | 5'GGCAATTGGCTAGGTTGTTCATG3' |
| mDell-3 F | 5'AGTTGCACTTCTCCTACCGCG3' |
| mDell-3 R | 5'ACGGCATTCATCAGGCTCTTC3' |
| mDell-4 F | 5'GTGAACTGCACATCAGCGATTG3' |
| mDell-4 R | 5'GTTGCAGACGAAGTTGTTTGGG3' |
| hNotch4 F | 5'TGTGAACGTGATGTCAACGAG3' |
| hNotch4 R | 5'ACAGTCTGGGCCTATGAAACC3' |
| hGAPDH F | 5'GGAGCGAGATCCCTCCAAAAT3' |
| hGAPDH R | 5'GGCTGTTGTCATACTTCTCATGG3' |

**Supplementary Table3. Allele frequencies of SNPs within genes in TB patients and controls**

| **Chr.** | **Gene** | **SNP** | **Allele**  **(MAF/minor)** | **Chr. Position** | **SNP Property** | **OR** | **95%CI** | **p**  **value** |
| --- | --- | --- | --- | --- | --- | --- | --- | --- |
| 1 | FASLG | rs763110 | C/T | 172627498 | 5'-flanking | 0.88 | 0.71~1.09 | 0.241 |
| 2 | NLRC4 | rs6757121 | C/T | 32449261 | 3'-flanking | 1.354 | 0.903～2.030 | 0.141 |
| 3 | IRAK2 | rs3844283 | C/G | 10264480 | exon9 | 1.089 | 0.837～1.417 | 0.526 |
| 4 | CXCL5 | rs3775488 | C/T | 74862852 | 3'-UTR(exon4) | 0.799 | 0.643～0.992 | 0.052 |
| 5 | CXCL14 | rs9327726 | C/T | 134915109 | 5'-flanking | 1.13 | 0.895~1.401 | 0.599 |
| 5 | IL4 | rs2070874 | C/T | 132009710 | exon1 | 0.865 | 0.678～1.104 | 0.244 |
| 6 | IRF4 | rs1050976 | C/T | 408079 | 3'-UTR(exon9) | 1.245 | 1.002～1.545 | 0.058 |
| 6 | IRF4 | rs7768807 | C/T | 408246 | 3'-UTR(exon9) | 1.077 | 0.85~1.263 | 0.278 |
| 6 | IRF4 | rs1050975 | A/G | 408012 | 3'-UTR(exon9) | 0.712 | 0.597~1.169 | 0.33 |
| 6 | NOTCH4 | rs422951 | A/G | 32188383 | exon6 | 0.725 | 0.564~0.933 | 0.012 |
| 6 | NOTCH4 | rs115244411 | A/G | 32188640 | exon5 | 0.827 | 0.619～1.106 | 0.2 |
| 6 | NOTCH4 | rs520803 | C/T | 32188603 | exon5 | 0.853 | 0.637～1.142 | 0.286 |
| 6 | NOTCH4 | rs114346832 | A/C | 32190390 | exon3 | 0.967 | 0.795～1.177 | 0.741 |
| 6 | NOTCH4 | rs206018 | C/G | 32154982 | intron | 0.777 | 0.619~0.976 | 0.047 |
| 7 | NOD1 | rs2906766 | C/T | 30499575 | 5'-UTR(exon2) | 0.865 | 0.704～1.063 | 0.167 |
| 7 | NOD1 | rs2075820 | A/G | 30492237 | exon6 | 0.955 | 0.779~1.129 | 0.075 |
| 7 | NOD1 | rs2736726 | A/G | 30519030 | 5'-flanking | 0.865 | 0.704～1.063 | 0.167 |
| 8 | RIPK2 | rs40457 | A/G | 90823687 | 3'-flanking | 0.873 | 0.698～1.091 | 0.232 |
| 8 | RIPK2 | rs16900627 | A/G | 90802995 | 3'-UTR(exon11) | 0.894 | 0.699~1.113 | 0.905 |
| 8 | RIPK2 | rs42490 | A/G | 90778513 | intron3 | 0.968 | 0.793～1.18 | 0.744 |
| 9 | IFNB1 | rs1424855 | C/G | 21078815 | 5'-flanking | 0.799 | 0.634～1.006 | 0.056 |
| 9 | IFNB1 | rs9333358 | A/G | 21078952 | 5'-flanking | 0.897 | 0.717～1.121 | 0.332 |
| 9 | LCN2 | rs12336742 | C/T | 130916041 | 5'-flanking | 0.95 | 0.781～1.157 | 0.612 |
| 9 | LCN2 | rs3814526 | A/G | 130910687 | 5'-flanking | 1.101 | 0.903～1.343 | 0.341 |
| 9 | TNFSF15 | rs10114470 | C/T | 117547772 | 3'-UTR | 0.953 | 0.783～1.159 | 0.628 |
| 9 | TNFSF15 | rs4574921 | C/T | 117538334 | 3'-flanking | 1.19 | 0.985~1.304 | 0.72 |
| 9 | TNFSF15 | rs6478108 | C/T | 117558703 | intron2 | 1.016 | 0.834～1.237 | 0.877 |
| 9 | PDCD1LG2 | rs7854413 | C/T | 5557708 | exon5 | 1.032 | 0.77～1.39 | 0.506 |
| 9 | PDCD1LG2 | rs16923189 | A/G | 5510644 | 5'-UTR(exon1) | 1.054 | 0.808～1.376 | 0.698 |
| 9 | CARD9 | rs4077515 | A/G | 139266496 | exon2 | 0.997 | 0.805～1.179 | 0.641 |
| 10 | GATA3 | rs10905277 | A/G | 8097368 | 5'-UTR(exon2) | 0.902 | 0.733～1.107 | 0.325 |
| 11 | TRAF6 | rs5030411 | C/T | 36533753 | 5'-flanking | 1.129 | 0.909～1.403 | 0.273 |
| 11 | TRAF6 | rs5030416 | A/C | 36532488 | 5'-flanking | 0.97 | 0.72～1.305 | 0.839 |
| 12 | LTA4H | rs17525495 | C/T | 96429377 | 5'-flanking | 0.911 | 0.734～1.131 | 0.398 |
| 12 | LTA4H | rs1978331 | C/T | 96409201 | intron | 0.962 | 0.789～1.175 | 0.706 |
| 12 | LTA4H | rs2540487 | A/G | 96429783 | 5'-flanking | 1.13 | 0.87～1.39 | 0.936 |
| 17 | CCL11 | rs17809012 | A/G | 32612444 | 5'-flanking | 0.83 | 0.674～1.022 | 0.079 |
| 17 | CCL11 | rs17735961 | A/C | 32612340 | 5'-flanking | 0.795 | 0.606～1.041 | 0.095 |
| 17 | CCL11 | rs4795896 | C/T | 32612252 | 5'-flanking | 0.904 | 0.741～1.104 | 0.324 |
| 17 | ARRB2 | rs2036657 | A/G | 4625159 | 3'-flanking | 0.965 | 0.688～1.114 | 0.244 |
| 17 | NLRP1 | rs925595 | C/G | 5488065 | 5'-flanking | 1.117 | 0.859～1.451 | 0.409 |

CI, confidence interval; OR, odds ratio; OR referring to the minor allele，MAF: major allele frequency

**Supplementary Table 2. Primer sequences**

| qPCR primers |  |
| --- | --- |
| mGAPDH F | 5'CCCACTAACATCAAATGGGG3' |
| mGAPDH R | 5'CCTTCCACAATGCCAAAGTT3' |
| mNotch4 F | 5'CTCTTGCCACTCAATTTCCCT3' |
| mNotch4 R | 5'TTGCAGAGTTGGGTATCCCTG3' |
| mJagged1 F | 5'GAAGTCAGAGTTCAGAGGCGTCC3' |
| mJagged1 R | 5'AGTAGAAGGCTGTCACCAAGCAAC3' |
| mJagged2 F | 5'TGCTGTGGAGGTGGCTATGTCT3' |
| mJagged2 R | 5'TGTTTCCACCTTGACCTCGGT3' |
| mDell-1 F | 5'GGACCTCATGAGGCATATGG3' |
| mDell-1 R | 5'GGCAATTGGCTAGGTTGTTCATG3' |
| mDell-3 F | 5'AGTTGCACTTCTCCTACCGCG3' |
| mDell-3 R | 5'ACGGCATTCATCAGGCTCTTC3' |
| mDell-4 F | 5'GTGAACTGCACATCAGCGATTG3' |
| mDell-4 R | 5'GTTGCAGACGAAGTTGTTTGGG3' |
| hNotch4 F | 5'TGTGAACGTGATGTCAACGAG3' |
| hNotch4 R | 5'ACAGTCTGGGCCTATGAAACC3' |
| hGAPDH F | 5'GGAGCGAGATCCCTCCAAAAT3' |
| hGAPDH R | 5'GGCTGTTGTCATACTTCTCATGG3' |
